# Human Myeloid-Derived Growth Factor Induces a Pro-Angiogenic Response and Functional Recovery in a Mouse Model of Peripheral Artery Disease

**DOI:** 10.3390/ijms27156903

**Published:** 2026-08-01

**Authors:** Anton Pekcec, Maria Myzithras, Cornelia Walther, Thomas Ciossek

**Affiliations:** 1 Boehringer Ingelheim Pharma GmbH & Co KG, Birkendorfer Str. 65, 88400 Biberach an der Riss, Germany; thomas.ciossek@boehringer-ingelheim.com; 2 Boehringer Ingelheim Pharmaceuticals, 900 Ridgebury Road, Ridgefield, CT 06877, USA; maria.myzithras@boehringer-ingelheim.com; 3 Boehringer Ingelheim RCV GmbH & Co KG, Dr.-Boehringer-Gasse 5–11, 1120 Vienna, Austria; cornelia.walther@boehringer-ingelheim.com

**Keywords:** human myeloid-derived growth factor, peripheral artery disease, mouse hindlimb ischemia model, perfusion, angiogenesis

## Abstract

Myeloid-derived growth factor (MYDGF) is a monocyte- and macrophage-secreted protein with anti-apoptotic and pro-angiogenic properties that has demonstrated protective effects in models of myocardial ischemia, but its role in peripheral ischemic injury has not been evaluated. We investigated the effects of recombinant human MYDGF (hMYDGF) in a murine hindlimb ischemia model. Male C57BL/6 mice underwent femoral artery ligation and were treated with continuous subcutaneous infusion of recombinant hMYDGF, vehicle control, or vascular endothelial growth factor (VEGF) as a positive control. Limb perfusion was assessed longitudinally using laser speckle contrast imaging, and functional recovery was evaluated using standardized limb function scoring. Recombinant hMYDGF significantly improved blood flow recovery in the ischemic limb compared with vehicle at all post-surgical time points, achieving levels of perfusion comparable to VEGF. Improved perfusion translated into accelerated early functional recovery, with a greater proportion of recombinant hMYDGF-treated mice retaining normal toe flexion. Immunohistochemical analyses revealed significantly increased CD34^+^ endothelial cell staining in both quadriceps and gastrocnemius muscles in recombinant hMYDGF-treated mice, consistent with enhanced angiogenesis, while alpha-smooth muscle actin staining did not differ between groups. Collectively, these findings demonstrate that recombinant hMYDGF restores blood flow and accelerates functional recovery following ischemic injury, supporting its therapeutic potential for ischemic diseases.

## 1. Introduction

Myeloid-derived growth factor (MYDGF), encoded by an open reading frame on chromosome 19 (C19orf10), is a novel protein secreted by bone marrow-derived monocytes and macrophages that has strong anti-apoptotic and tissue repair properties, including pro-angiogenic effects [1,2,3].

MYDGF was initially characterized in the context of cardioprotection and myocardial repair following myocardial infarction (MI) [1]. Subsequent studies have expanded its relevance beyond cardiac injury, providing rationale for MYDGF in the treatment of a broad range of pathologic conditions, including atherosclerosis and heart failure, metabolic disorders, kidney disease, autoimmune and inflammatory diseases [2]. A recent systematic review of protein-based therapies for acute MI identified several recombinant proteins capable of promoting cardiac repair and improving cardiac function through pleiotropic mechanisms, with MYDGF demonstrating consistent and robust effects across multiple outcomes and models [4].

*Mydgf*-deficient mice have been shown to develop larger infarct size post-MI, higher levels of cardiomyocyte apoptosis, reduced endothelial proliferation, and impaired angiogenesis compared with wildtype mice. Notably, administration of recombinant MYDGF to *Mydgf*-deficient mice attenuates these effects, reducing infarct size and contractile dysfunction [1].

The pro-angiogenic effects of MYDGF have been demonstrated on primary endothelial cells in models of tissue ischemia [5]. Studies have indicated that MYDGF stimulates endothelial and epithelial cell proliferation through phosphorylation of MAPK1/3 and STAT3 signaling [6,7]. Beyond its vascular effects, MYDGF has also been shown to promote cardiomyocyte proliferation and cardiac regeneration following MI in neonatal and adult mice [8]. Currently, recombinant human MYDGF (BI 765845) is being evaluated in clinical trials of cardio-renal-metabolic conditions.

Investigating the potential of MYDGF to induce angiogenesis and enhance functional recovery after ischemia has implications for the treatment of ischemia-reperfusion injury following MI. The mouse hindlimb ischemia (HLI) model is a widely used preclinical model for investigating peripheral arterial disease and critical limb ischemia. This model is often used to investigate the potential of new treatments to induce neovascularization [9]; however, to date, MYDGF has not been assessed in this model.

In this study, we assessed the effects of recombinant human (hMYDGF) on blood flow and functional recovery after induced ischemia in a mouse HLI model.

## 2. Results

### 2.1. Mortality and Body Weight

A total of four mice (8.9%) did not complete the study. One mouse from group 3 died during the surgical procedure on Day 1, two mice from group 2 were euthanized for humane reasons on Day 2, and one mouse from group 1 was found dead in its cage on Day 2.

Body weight was monitored through Day 29. All groups experienced post-surgical weight loss but recovered to near-baseline levels by Day 29. Mean body weight (standard deviation) at Day 29 was 23.6 (1.0) g for group 1, 23.1 (1.1) g for group 2, and 24.3 (1.8) g for group 3. Longitudinal body weight measurements were similar across groups (Table 1).

No statistically significant differences in body weight were observed between groups over the course of the study, with the exception of a single difference (*p* < 0.05) between the recombinant hMYDGF and VEGF groups on Day 22 (Figure S1).

### 2.2. Blood Flow and Limb Function Improvements

**Figure 1:**
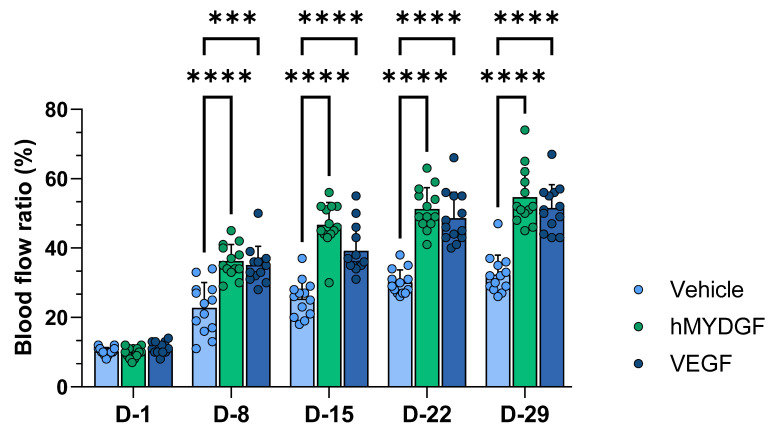
Blood flow ratio (%) was measured at indicated time points in mice treated with Vehicle, recombinant hMYDGF, or VEGF. Data show mean ± SEM with individual data points. Statistics: two-way repeated measures ANOVA with Dunnett’s multiple comparisons test vs. Vehicle. *** *p* < 0.001, **** *p* < 0.0001, Vehicle (*n* = 12), hMYDGF (*n* = 11), VEGF (*n* = 13). D: Day; hMYDGF: human myeloid-derived growth factor; SEM: standard error of the mean; VEGF: vascular endothelial growth factor.

Statistically significant improvements (*p* < 0.001) in blood flow were observed in the recombinant hMYDGF and VEGF groups compared with vehicle at Days 8, 15, 22, and 29 (Figure 1).

**Figure 2:**
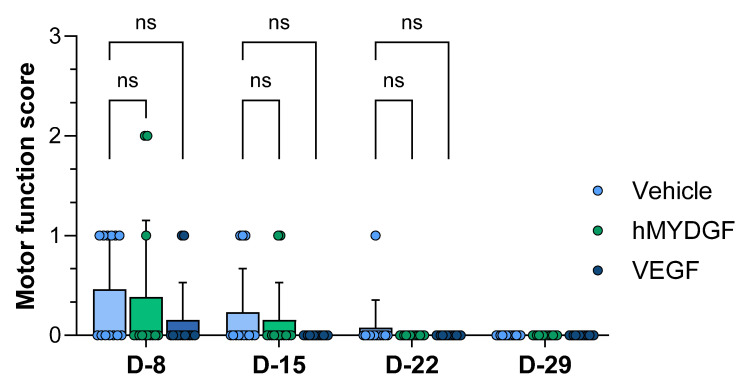
Motor function score over time: Motor function score (0–3) was assessed at Days 8, 15, 22, and 29 in mice treated with Vehicle, hMYDGF, or VEGF. Vehicle (*n* = 12), hMYDGF (*n* = 11), VEGF (*n* = 13). Data are shown as mean ± SEM with individual data points. Statistical analysis was performed using two-way repeated measures ANOVA followed by Bonferroni’s multiple comparisons test, comparing each treatment group to Vehicle at each time point. ns: not significant.

Improvements in limb function were observed across all groups (Figure 2). On Day 8, animals treated with recombinant hMYDGF or VEGF demonstrated greater functional recovery than vehicle-treated animals, with 76.9% and 85.7% of animals, respectively, achieving a functional score of 0 (preserved ability to flex the toes to resist gentle traction of the tail), compared with 53.9% in the vehicle group. All animals had full recovery of limb function (score of 0) by Day 29.

### 2.3. Histologic Findings

No foot amputations (Grades 2–4) were observed in any treatment group, and toe necrosis (Grade 1) was identified in only one animal from the vehicle group.

Generally, atrophy lesions were mild, characterized by focal atrophy and by mild diffuse inflammation, and composed mainly of lymphocytes; satellite cells were almost not observed.

In the quadriceps muscle, mean atrophy scores ranged from 1.36 to 1.54 (indicating minor muscle atrophy, i.e., up to 10% of muscle fibers), while inflammatory scores ranged between 1.23 and 1.29 (indicating mild cellular infiltration, with an increase of up to 10 cells per ×20 magnification) (Tables S1 and S2). Compared with their respective controls (non-injured hindlimbs), significant differences were observed in atrophy scores for groups 1 (*p* = 0.023) and 2 (*p* = 0.05), as well as in inflammatory scores for all groups (*p* = 0.003, *p* = 0.021, and *p* = 0.021).

In the gastrocnemius muscle, atrophy scores ranged from 1.54 to 1.79 and inflammatory scores from 1.23 to 1.46 across groups (Table S2). The differences between each group and its control were significant in all groups (*p* < 0.05).

Representative H&E-stained sections illustrating the mild degree of muscle atrophy and inflammatory cell infiltration in the ischemic hindlimb compared with the corresponding non-injured control are shown in Figure S2A,B.

### 2.4. Digital Quantitative Morphometry

**Figure 3:**
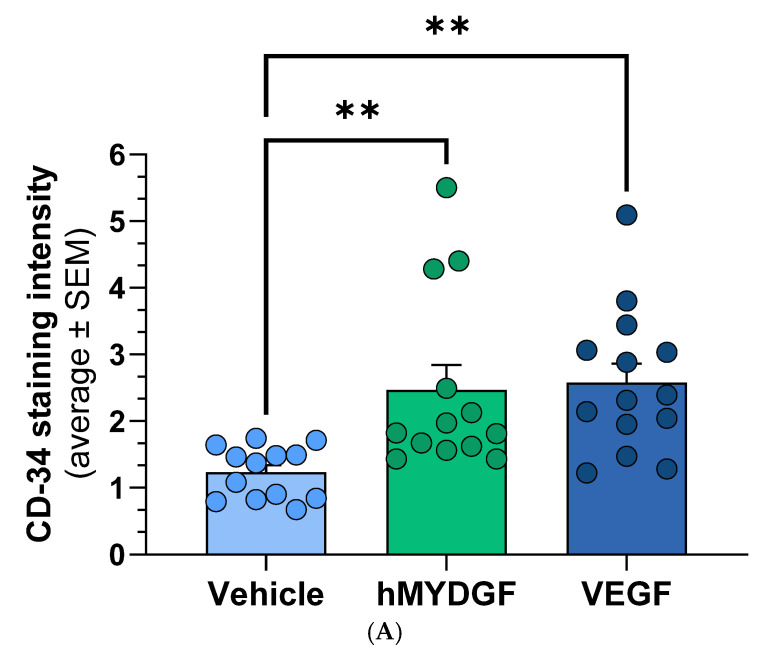

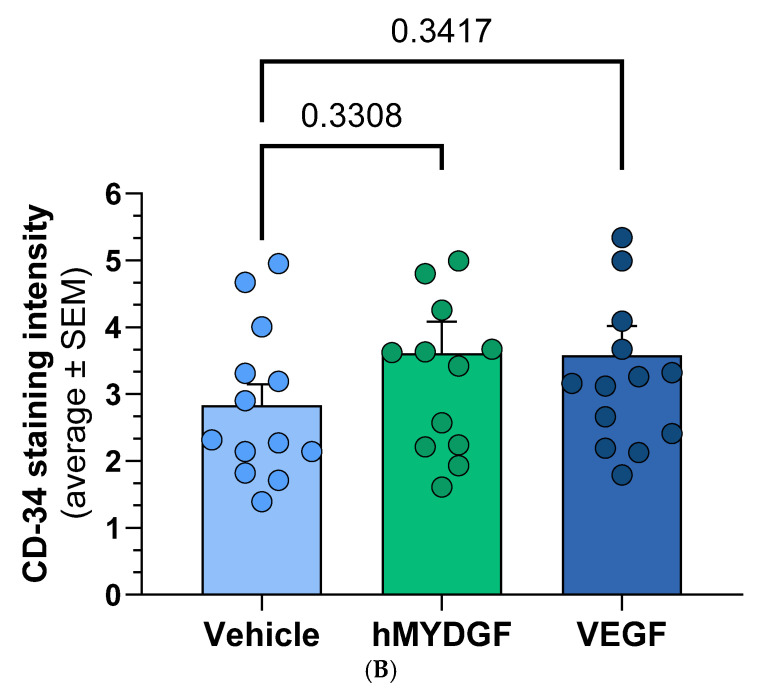
(**A**) CD34^+^ evaluation in quadriceps (Day 29). (**B**) CD34^+^ evaluation in gastrocnemius (Day 29). CD34^+^ staining intensity (% area) was quantified in quadriceps and gastrocnemius muscle sections from mice treated with vehicle (Vehicle), recombinant hMYDGF, or VEGF on Day 29. Each symbol represents an individual animal, and bars indicate the mean ± SEM. Vehicle (*n* = 12), hMYDGF (*n* = 11), VEGF (*n* = 13). Data were analyzed using one-way ANOVA followed by Tukey’s multiple comparisons test. ** *p* < 0.01, and bottom panel: Dunnett’s multiple comparison test. hMYDGF: human myeloid-derived growth factor; SEM: standard error of the mean; VEGF: vascular endothelial growth factor.

CD34^+^ staining in the quadriceps muscle showed a significantly greater stained area (*p* < 0.01) in groups 2 and 3 (2.5% and 2.6%, respectively) compared with group 1 (1.2%) (Figure 3A). A similar pattern was observed in the gastrocnemius muscle, where the stained area was higher in groups 2 and 3 (3.6% and 3.6%, respectively) than in group 1 (2.8%), although this did not reach significance (Figure 3B). Representative CD34^+^ immunohistochemical staining is shown in Figure S2C,D. In contrast, alpha-smooth muscle actin (α-SMA) staining did not differ between groups in either muscle analyzed, with representative α-SMA immunohistochemical images presented in Figure S2E,F.

### 2.5. Recombinant hMYDGF Concentrations in Mouse Plasma

Overall, MYDGF exposure was consistent with drug concentration, infusion rate, and duration, supporting successful administration of recombinant hMYDGF during the study (Table 2 and Table S3). The dose was based on published literature, previously suggested to be efficacious [1,10], and calculated using MYDGF concentration and molecular-weight-adjusted clearance [11], assuming steady-state infusion.

## 3. Discussion

In this murine model of HLI, SC administration of recombinant hMYDGF improved blood flow in the injured (ischemic) limb of C57/BL mice, as measured using laser speckle contrast imaging. Recombinant hMYDGF significantly increased blood perfusion compared with vehicle-treated control from Day 8. Blood perfusion in the recombinant hMYDGF group was numerically similar to that observed in the VEGF-treated group (positive control); however, direct comparisons should be interpreted cautiously as recombinant hMYDGF and VEGF were administered using different routes and dosing schedules.

Improved perfusion was accompanied by accelerated early functional recovery; 77% of mice treated with recombinant hMYDGF retained the ability to flex their toes in response to gentle traction of the tail (similar to the VEGF group: 86%) at Day 8; however, direct comparisons are limited due to the differences in administration route and schedule between treatments. The perfusion findings were further supported by immunohistochemistry (IHC) analyses: CD34^+^ staining in the operated hind limb was of significantly higher intensity in the recombinant hMYDGF group (similar to the VEGF group) versus vehicle; however, this was not seen in the gastrocnemius muscle section. While CD34^+^ staining is consistent with increased vascularization and endothelial cell presence within ischemic tissue, the present study did not directly assess endothelial proliferation, vessel maturation, or MYDGF-related signaling pathways. Moreover, α-SMA staining was not significantly different between treatment groups. As α-SMA is commonly used as a marker of vascular smooth muscle cells and more mature vessels, the absence of a corresponding increase suggests that the enhanced CD34^+^ staining may primarily reflect increased endothelial cell presence rather than arteriogenesis or formation of mature functional vessels. Therefore, these findings should be interpreted as supportive, but not definitive, evidence of angiogenic activity.

The present findings support a model in which MYDGF enhances recovery from ischemic injury primarily through endothelial-supportive and pro-vascular mechanisms. MYDGF has previously been shown to promote endothelial proliferation, migration, survival and angiogenesis through MAPK/STAT3 signaling and fibroblast growth factor (FGF)-1-dependent pathways [5,6,7] and improves neovascularization and cardiac dysfunction in MI and ischemia-reperfusion models [1,5,12]. In the current study, recombinant hMYDGF treatment was associated with improved perfusion recovery, enhanced functional recovery, and increased CD34^+^ staining in ischemic skeletal muscle. These findings are consistent with the endothelial-supportive and pro-vascular effects reported previously [5]. Although the present observations are consistent with activation of previously described MYDGF-mediated pathways [6,7], direct evidence linking MYDGF to endothelial proliferation, vessel maturation, or FGF1 signaling in skeletal muscle was not obtained and remains an important area for future investigation.

There is increasing interest in the therapeutic potential of MYDGF in ischemia and, in particular, ischemia-reperfusion injury. First characterized in a 2015 bioinformatic secretome analysis, MYDGF was identified as an endogenous protein secreted by monocytes and macrophages derived from the bone marrow [1,2]. In the same study, knockout mice lacking the *Mydgf* gene had larger infarct scars after induced MI and more severe contractile dysfunction, and these effects were attenuated after administration of recombinant MYDGF [1]. Subsequent studies conducted in *Mydgf* knockout mice have reported similar findings in terms of improvements in scar size, systolic function, and heart regeneration after administration of recombinant MYDGF [8,13].

The growing interest in MYDGF as a therapeutic candidate is reflected in a recent systematic review of protein-based therapies for MI and ischemia–reperfusion injury, which identified MYDGF as one of the most promising agents for ischemic tissue repair. The review highlighted improvements across preclinical models, including reductions in infarct size and fibrosis, together with enhanced angiogenesis, cardiac function, and survival, while also emphasizing the challenges associated with translating these findings into clinical benefit [4].

Importantly, translational pharmacokinetic modelling has suggested that the exposure associated with efficacy in preclinical MI models may be achieved in humans at clinically feasible dose levels [10]. These findings could potentially provide support for the application of MYDGF in humans and could provide a rationale for further clinical evaluation to determine whether the cardioprotective effects observed in preclinical studies translate to patients. However, these translational considerations should be interpreted cautiously. The present findings were obtained in young healthy male mice using an acute hindlimb ischemia model, which only partially recapitulates the complex pathophysiology of peripheral artery disease and has historically shown limited predictive value for clinical efficacy of pro-angiogenic therapies.

Other angiogenic compounds and chemokines have been tested in the HLI model, including VEGF-A (used as a positive control in this study), hepatocyte growth factor (HGF), FGF-2, basic FGF, stromal-derived factor-1 alpha (SDF-1α; also known as CXCL12), and platelet-derived growth factor-BB (PDGF-BB).

In one study, intramuscular administration of VEGF165 (delivered via adeno-associated viral vector) stimulated angiogenesis and improved blood flow in a rat HLI model [14]. In another rat and rabbit HLI model, VEGF-A and heme oxygenase-1-modified endothelial progenitor cells synergized with each other in promoting angiogenesis in the ischemic limb [15]. Similarly, intramuscular injection of human HGF via plasmid delivery induced therapeutic angiogenesis in both rat and rabbit HLI models of ischemia [16]. Additionally, recombinant HGF induced angiogenesis in a rabbit model of HLI [17].

Administration of basic FGF-2 in vitro has been shown to increase blood flow and capillary density in mice [18] and rabbits [19], and intramuscular injection of plasmid DNA containing FGF-2 and PDGF-BB promoted effective angiogenesis in mouse HLI [20]. In addition, the chemokine SDF-1α has shown potent effects on neovascularization and blood flow in mouse and rat HLI models [21,22], as well as improved muscle regeneration versus control in one study of mesenchymal stem cells engineered to secrete SDF-1α in a mouse model of HLI [23].

Several therapies (including growth factors such as VEGF) have failed clinical trials despite showing promise in the HLI model, highlighting the need for caution in interpreting findings from this model [24,25]. A detailed comparison between the present study and other HLI model studies is provided in Table S4.

This study has several limitations that should be considered when interpreting the results. Although blood flow was significantly improved with therapeutic intervention, vascular permeability was not assessed, and mechanistic endpoints relevant to angiogenesis were not evaluated. Although increased CD34^+^ staining and improved perfusion were observed following recombinant hMYDGF treatment, the study did not assess endothelial proliferation, capillary density, vessel maturity, pericyte coverage, or downstream signaling pathways associated with MYDGF activity. In addition, the lack of a significant difference in α-SMA staining limits conclusions regarding arteriogenesis or mature vessel formation. Future studies incorporating these endpoints would help clarify the mechanisms underlying the observed vascular and functional improvements. Furthermore, only male mice were used; all animals were young and healthy, and no dose–response evaluation of the test item was performed. The surgical method involved ligation of the femoral artery, a standard technique in the HLI model. A previous study found differences in ischemic severity depending on surgical method, which can affect functional recovery and interpretation of treatment effects [26]. Interestingly, reduced blood flow perfusion following HLI induction and during follow-up may depend on the strain of mouse [26]. A study evaluating post-natal angiogenesis in a mouse HLI model showed increased ischemia-induced angiogenic capacities in C57BL/6J mice compared with SV-129 mice, which developed aggravated tissue damage, inflammation, and impaired angiogenesis [27]. This should be kept in mind when interpreting the results of this study, which used C57/BL mice. In line with this, Recatalá et al. (2023) developed a study protocol for the HLI model that includes the use of four different mouse strains (BALB/cJ, CD-1 IGS, 129/Sv, and C57BL/6J), specifically to allow comparisons of post-ischemic responses across genetic backgrounds [28]. In addition, the HLI model is characterized by pathophysiological mechanisms that differ from, or only partially recapitulate, those observed in patients with critical limb ischemia, the transient nature of the ischemic insult, and a heightened responsiveness to therapies that have not demonstrated efficacy in clinical settings [29,30]. Accordingly, the results should be regarded as model-specific and hypothesis-generating, with uncertain translatability to clinical disease. Finally, the follow-up in the present study was limited to 29 days; future studies may assess longer-term outcomes.

## 4. Materials and Methods

### 4.1. Ethical Statement

Animal handling was performed according to guidelines of the US National Institutes of Health and the Association for Assessment and Accreditation of Laboratory Animal Care. Ethical approval was granted by the Israel Board for Animal Experiments and in compliance with the Israel Animal Welfare Act (approval number: IL-20-8-390). The study was conducted by PharmaSeed Ltd. (Ness Ziona, Israel).

The results are reported in accordance with the ARRIVE guidelines (Animal Research: Reporting of In Vivo Experiments) [31].

### 4.2. Study Design

A total of 45 C57/BL mice were used in this study. Animals were equally divided into three treatment groups prior to HLI surgery: vehicle control (group 1, *n* = 13); recombinant hMYDGF test compound (group 2, *n* = 13); and vascular endothelial growth factor (VEGF) pro-angiogenic positive control (group 3, *n* = 14).

Recombinant hMYDGF was produced using *Escherichia coli-*fed batch fermentation. Following fermentation, bacterial biomass was harvested by centrifugation. Inclusion bodies were recovered by high-pressure homogenization and subsequent centrifugation using a tubular bowl centrifuge.

The isolated inclusion bodies were solubilized under denaturing conditions and refolded to restore the native protein conformation. The refolded solution was subjected to ultrafiltration and diafiltration prior to capture using anion exchange chromatography. Final purification was achieved by hydrophobic interaction chromatography. The purified protein solution was subsequently ultra- and diafiltrated, concentrated, and formulated to a final concentration of 10 g/L in 20 mM HEPES, 100 mM NaCl, pH 7.0. The final solution contained 4–5% of methionine truncations based on mass spectrometry measurements.

### 4.3. Experimental Animals

Male C57/BL/C mice aged 7–8 weeks were obtained from Envigo RMS Ltd. (Ness Ziona, Israel). Only male mice were used in order to eliminate sex as a potential confounding variable in the assessment of treatment effects. Mice were specific pathogen-free and treatment-naïve. No criteria for including and excluding animals were set. Mice were allocated into study groups according to their body weight before surgery to ensure balance across the treatment arms. The individual body weights of the mice were in the range of ±20% of the group’s mean. The mice were fed ad libitum on a commercial rodent diet, with free access to autoclaved and acidified drinking water (pH 2.5–3.5), and were housed in a climate-controlled environment (18–24 °C, relative humidity range 30–70%, 12 h light/dark cycle).

### 4.4. Experimental Procedures

#### 4.4.1. HLI Procedure

The study was conducted in PharmaSeed Ltd. facilities in Ness Ziona, Israel. Animals in groups 1 and 2 were treated by continuous subcutaneous (SC) infusion using implanted iPRECIO SMP-310R pumps for 4 weeks, starting 1 day prior to surgery. Group 3 was treated with VEGF by local intramuscular injection of 3.3 µg/100 µL at two anatomical sites per animal on Day 2. SC infusion was selected as the route of administration as this allows a drug exposure that has previously been shown to drive angiogenesis in another mouse in vivo study [1].

Mice were anesthetized with isoflurane (1.5%) and placed ventral side up. Analgesia was provided with meloxicam (2 mg/kg, SC), administered 1 h prior to HLI surgery on Day 1 and once daily for the subsequent 2 days. A skin incision was made in the inguinal region of the right hind limb, and HLI was induced as previously described [32]. Following the procedure, the surgical wound was closed using 5-0 Vicryl absorbable thread. Postoperative analgesia was provided with buprenorphine (0.02 mg/kg) administered twice daily during the 2 days following surgery.

Animals underwent an acclimation period of 15–18 days prior to study initiation, and were monitored for 29 days following HLI surgery. Body weight, mortality, limb function, necrosis, and blood flow were assessed throughout the study period. Functional assessments, including limb function scoring and blood flow measurements, were performed by an investigator blinded to treatment allocation. A detailed study timeline and description of additional procedures and evaluations are provided in Table S5.

#### 4.4.2. Blood Flow and Limb Function Measurement

Blood flow in both hind legs for each mouse was measured with a non-contact Speckle Doppler before surgery at baseline and after surgery on Days 8, 15, 22, and 29. Only animals with a ≥30% reduction in blood flow in the ischemic leg compared with the uninjured leg were included in the study.

Limb function impairment of the ischemic limb was assessed semi-quantitatively twice weekly following surgery, beginning on Day 8 and subsequently on Days 15, 22, and 29. Functional scoring was performed according to a previously described scale [33]: a score of 0 indicated flexion of the toes in response to gentle tail traction; 1 indicated plantar flexion; 2 indicated absence of plantar flexion without foot dragging; and 3 indicated dragging of the foot.

#### 4.4.3. Histologic Analysis

Quadriceps and gastrocnemius muscle tissues were harvested from 40 mice, fixed in 4% formaldehyde, and transferred to Patho-Logica for histopathologic evaluation. Tissues were maintained in fixative for 48 h prior to processing. Samples were trimmed, placed into embedding cassettes (one cassette per animal), and processed using standard protocols for paraffin embedding. Paraffin sections (4 μm thickness) were prepared, mounted on glass slides, and stained with hematoxylin and eosin (H&E) for general histologic assessment.

Histologic images were acquired using an Olympus BX60 microscope (serial no. 7D04032, Tokyo, Japan) equipped with an Olympus DP73 camera (serial no. OH05504, Tokyo, Japan), at ×10 and ×20 objective magnifications. Representative images were collected from each experimental group for analysis.

Macroscopic evaluation of the ischemic hind limb was performed twice weekly following surgery and increased to twice daily after the initial appearance of necrosis. Limb necrosis was graded beginning on Day 8 post-operation and subsequently on Days 15, 22, and 29 using a previously described morphologic scoring system as follows [34]:

0, no necrosis; 1, necrosis limited to the toes (toe loss); 2, necrosis extending to the dorsum pedis (foot loss); 3, necrosis extending to the crus (knee loss); and 4, necrosis extending to the thigh (complete hind limb loss).

Semi-quantitative assessment of muscle atrophy, based on H&E staining, and inflammation, based on the extent of cellular infiltration by macrophages and satellite cells, was performed using a 0–4 scoring scale as detailed in Table S2.

IHC was performed for the detection of CD34^+^ (endothelial cells) and alpha-smooth muscle actin (α-SMA) (blood vessels); all slides were subjected to histopathologic evaluation by an independent adjudicator. Quantitative analysis of IHC staining was performed using a computerized image analysis with the Image Pro Plus software version 6.0. Results were presented as the percentage of the whole slide area stained positive for either CD34^+^ (endothelial cells) or α-SMA (number of blood vessels counted).

### 4.5. Outcome Measures

The outcomes assessed during the study included morbidity and mortality, which were monitored twice daily, and changes in body weight recorded through Day 29. Limb perfusion was evaluated up to Day 29 by calculating the blood flow ratio between the right and left hind limbs using Speckle Doppler imaging. Ischemic severity was assessed macroscopically between Days 8 and 29, together with semi-quantitative evaluation of limb function over the same period.

Histologic outcome measures included semi-quantitative scoring of muscle atrophy and inflammation in quadriceps and gastrocnemius muscles, and were further evaluated by digital quantitative morphometric analysis for CD34^+^ cells and α-SMA.

### 4.6. Statistical Analysis

The number of groups and the total number of animals were based on previous studies demonstrating that this was the minimum number of animals per group sufficient to obtain indicative/significant information.

Body weight and blood flow data were analyzed using GraphPad Prism version 5.02. Body weight data were analyzed using a two-way analysis of variance ANOVA.

Blood flow data were analyzed using a two-way repeated-measures ANOVA, with treatment as the between-subject factor and time of blood flow assessment as the within-subject (repeated-measures) factor.

Post hoc comparisons versus the vehicle-treated group were performed using Bonferroni’s multiple-comparisons test to account for multiple testing. Animals that did not complete the study (e.g., due to death or missing measurements) were excluded from the repeated-measures analysis, as a complete longitudinal dataset was required.

One-way ANOVA followed by Bonferroni post hoc comparisons (GraphPad Prism 5.02) was conducted for the CD34^+^ quadriceps evaluation; a two-tailed *t*-test was used for the CD34^+^ evaluation in the gastrocnemius muscle.

## 5. Conclusions

This study evaluated the angiogenic efficacy of recombinant hMYDGF in a murine HLI model and demonstrated that recombinant hMYDGF significantly improved blood flow compared with vehicle, achieving levels of perfusion recovery comparable to those observed with VEGF. These functional improvements were supported by IHC analyses of the operated hind limbs, which revealed significantly increased CD34^+^ staining relative to control, consistent with enhanced vascularization in ischemic tissue. However, additional mechanistic studies are required to establish whether recombinant hMYDGF directly promotes angiogenesis and to define the cellular and molecular pathways involved. Collectively, these findings highlight the therapeutic potential of recombinant hMYDGF in a murine hindlimb ischemia model. These results provide a potential basis for further investigation of MYDGF in ischemic injury, and the ongoing clinical evaluation of recombinant hMYDGF (BI 765845) in cardio-renal-metabolic conditions.

## Figures and Tables

**Table 1:** Mean (standard deviation) body weight (g) measurements by group during the study.

Group	Day 1	Day 8	Day 15	Day 22	Day 29
1 (vehicle)	21.8 (1.1)	21.2 (0.8)	23.0 (0.7)	23.5 (0.9)	23.6 (1.0)
2 (hMYDGF)	22.4 (1.1)	21.0 (1.3)	22.9 (1.3)	22.9 (1.1)	23.1 (1.1)
3 (VEGF)	22.5 (1.3)	22.1 (1.5)	23.3 (1.6)	24.4 (1.9)	24.3 (1.8)

hMYDGF: human myeloid-derived growth factor; VEGF: vascular endothelial growth factor.

**Table 2:** Recombinant hMYDGF mean (SD) concentrations in mouse plasma (ng/mL).

Animal	Baseline	2 Days	7 Days	14 Days	21 Days	28 Days
Mean	2865.46	2285.48	1795.71	4099.03	3366.12	60.56
SD	1092.19	1244.95	1184.61	1247.25	647.97	80.92

hMYDGF: human myeloid-derived growth factor.

## Data Availability

The data presented in this study are available on request from the corresponding author. The data are not publicly available due to privacy restrictions.

## Supplementary Materials

The following supporting information can be downloaded at: https://www.mdpi.com/article/10.3390/ijms27156903/s1. References [14,15,16,17,18,19,20,21,22,23,35] are cited in the Supplementary Materials.

## Abbreviations

The following abbreviations are used in this manuscript:

α-SMA	Alpha-smooth muscle actin
ANOVA	Analysis of variance
ARRIVE	Animal Research: Reporting of In Vivo Experiments
CMEC	Cardiac microvascular endothelial cell
FGF	Fibroblast growth factor
H&E	Hematoxylin and eosin
HGF	Hepatocyte growth factor
HLI	Hindlimb ischemia
hMYDGF	Human myeloid-derived growth factor
IHC	Immunohistochemistry
MI	Myocardial infarction
MYDGF	Myeloid-derived growth factor
PDGF-BB	Platelet-derived growth factor-BB
SC	Subcutaneous
SDF-1α	Stromal-derived factor-1 alpha
VEGF	Vascular endothelial growth factor
