# Supplementary material for: Human Myeloid-Derived Growth Factor Induces a Pro-Angiogenic Response and Functional Recovery in a Mouse Model of Peripheral Artery Disease
Source: Int J Mol Sci. 2026 Aug 1;27(15):6903. doi: 10.3390/ijms27156903 (PMC13467340; doi:10.3390/ijms27156903)
Supplement: Supplementary file 1 [file ijms-27-06903-s001.zip › ijms-4375271-SI.pdf]

Supplementary Materials

Table S1. Grading system for assessment of muscle atrophy and cellular inflammation.

**Score**	**Muscle atrophy (H&E staining)**
0	No atrophy
1	Minor (up to 10% of muscle fibers)
2	Mild (10–25% of muscle fibers)
3	Moderate (25–75% of muscle fibers)
4	Severe (75–100% of muscle fibers)
**Score**	**Cellular inflammation (macrophages and satellite cells)**
0	0 = No inflammatory infiltration
1	1 = Mild cellular infiltration with an increase of up to 10 cells per ×20 magnification
2	2 = Moderate cellular infiltration with an increase of 10–20 cells per ×20 magnification
3	3 = Severe cellular infiltration with an increase of 20–50 cells per ×20 magnification
4	4 = Enormous cellular infiltration with an increase of > 50 cells per ×20 magnification

H&E: hematoxylin and eosin.

Table S2. Histology results of quadriceps and gastrocnemius muscles by treatment group.

	**Quadriceps muscle**						**Gastrocnemius muscle**					
	1-Control $n = 3$	1 $n = 13$	2-Control $n = 3$	2 $n = 13$	3-Control $n = 3$	3 $n = 14$	1-Control $n = 3$	1 $n = 13$	2-Control $n = 3$	2 $n = 13$	3-Control $n = 3$	3 $n = 14$
Atrophy score; mean (SD)	0.33 (0.58)	1.38* (0.51)	0.67 (0.58)	1.54* (0.52)	0.67 (0.58)	1.36 (0.50)	0.33 (0.52)	1.54* (0.52)	0.67 (0.58)	1.54* (0.52)	0.33 (0.58)	1.79* (0.43)
Inflammation score; mean (SD)	0.33 (0.58)	1.23* (0.44)	0.33 (0.58)	1.23* (0.44)	0	1.29* (0.47)	0	1.46* (0.52)	0	1.46* (0.66)	0	1.29* (0.47)

* Indicates significant difference between a treatment group and its control ($p \leq 0.05$). The non-injured hindlimb was used as the control ($n = 3$ animals per group) and was assessed to determine whether the test compounds had any effect on the uninjured limb/tissue. SD: standard deviation.

Table S3. hMYDGF concentrations in mouse plasma (ng/mL).

Animal	Baseline	2 days	7 days	14 days	21 days	28 days
16	2838.85	4462.74	2359.04	3260.40	3311.28	53.59
17	5186.40	3400.91	15.76	5081.45	4235.44	BQL
18	2999.02	87.05	18.86	5767.82	3880.60	48.07
19	161.46	2441.45	2200.92	4078.93	3153.64	57.53
20	SNP	SNP	SNP	SNP	SNP	SNP
21	2723.32	2476.08	14.23	6705.24	4024.79	42.00
22	2773.12	2989.65	2414.34	4139.38	3474.63	4.08
23	3459.18	124.20	BQL	3555.24	2876.89	38.31
24	2184.03	2361.51	3707.49	2805.60	2229.08	29.06
25	2383.78	2263.93	1725.14	2524.34	2957.45	313.38
26	3453.58	3043.90	2430.33	3395.07	2768.85	24.31
27	3033.49	948.54	2209.56	2878.42	2721.29	29.24
28	3243.66	2416.77	1742.61	5087.89	4264.80	41.81
29	2811.11	2694.53	2710.24	4007.64	3860.83	45.29

BQL: below limit of quantitation (0.15ng/mL); hMYDGF: human myeloid-derived growth factor; SNP: sample not present.

Table S4. Comparative summary of present study versus other HLI model studies.

Study/model	Model	Therapy	Blood flow	Functional recovery	IHC/histology	Other
Present study	Mouse HLI	hMYDGF	Statistically significant improvements in the hMYDGF group compared with vehicle at Days 8, 15, 22, and 29	Improved limb function scores; higher proportion of animals with preserved toe flexion from Day 8	Increased CD34$^+$ endothelial area in quadriceps and gastrocnemius; no difference in α-SMA staining	N/A
Shimpo et al. 2026 [1]	Rat HLI	AAV-mediated VEGF gene transfer	The mean blood flow in AAV-VEGF-transduced ischemic limbs was significantly higher than in AAV-*LacZ*-transduced limbs	N/A	Capillary density was significantly higher in AAV-VEGF-injected tissues than in AAV-LacZ-injected tissues	RT-PCR, Western blotting, and ELISA showed sustained VEGF mRNA and protein expression in injected muscle for at least 10 weeks after AAV-VEGF delivery
Long et al. 2013 [2]	Rat HLI	VEGF-A- and HO-1-EPC transfer	Significantly improved perfusion recovery by laser Doppler imaging; greatest recovery in combined VEGF-A + HO-1 EPC group (~85% at Day 21 vs 28% PBS)	Significantly increased limb salvage rates; highest in VEGF-A + HO-1 EPC group (94.4%) compared with VEGF-A or HO-1 EPC alone and controls	Significantly increased the microvessel density (expressed as capillaries/m^2 at Day 21; greatest effect with combined VEGF-A + HO-1 EPCs	N/A
Taniyama et al. 2001 [3]	Rat and rabbit HLI	Intramuscular injection of naked human HGF plasmid	Significantly increased perfusion by laser Doppler imaging in rats; increased blood flow by Doppler flow wire and improved ischemic/normal limb blood pressure ratio in rabbits ($p < 0.01$)	N/A	Significantly increased capillary density in ischemic muscle in a dose-dependent manner; greater angiogenesis at 5 weeks compared with single recombinant HGF injection	N/A
Morishita et al. 1999 [4]	Rabbit HLI	rhHGF	Angiographic scores were significantly	N/A	N/A	N/A

			increased in rhHGF-treated animals vs vehicle ($p < 0.01$)			
Jin et al. 2021 [5]	Mouse HLI	bFGF delivered via fibrin-based ARSs with FUS-triggered release	Significantly increased perfusion over 4 weeks in the ARS + FUS group compared with control groups	Reduced visible tissue necrosis in the ARS + FUS group compared with other experimental groups	Significantly greater angiogenesis and reduced fibrosis in ischemic tissue in the ARS + FUS group	*In vitro* studies showed sustained, on-demand bFGF release with repeated FUS application
Horio et al. 2011 [6]	Rabbit HLI	F/P-MPs/FGF-2	Significant improvement in blood flow ratio and blood pressure ratio at Days 14 and 28 in the F/P-MPs/FGF-2 group compared with control, FGF-2 alone, and F/P-MPs alone	N/A	Increased capillary number at Day 28; higher numbers of $CD31^{+}$ endothelial and α-SMA^{+} vessels in the F/P-MPs/FGF-2 group compared with other groups	N/A
De Paula et al. 2009 [7]	Rat HLI	Nonviral plasmid-based dual gene transfer of FGF-2 and PDGF-BB	Improved perfusion consistent with enhanced angiogenesis and arteriogenesis; efficacy comparable to VEGF165-treated animals at 4 weeks	N/A	Increased angiogenesis and arteriogenesis; formation of more stable and mature vascular structures compared with VEGF165	Dual gene transfer enabled 50% lower individual plasmid doses; promoted more stable vascular networks than VEGF165, supporting improved vessel maturation
Edwards et al. 2017 [8]	Rat HLI	Intramuscular ESA with systemic GM-CSF	Significantly improved reperfusion ratios by laser Doppler imaging at Day 14 in ESA/GM-CSF-treated animals compared with saline/GM-CSF controls	N/A	Significantly increased capillary density in ischemic muscle at Day 14	Increased VEGF-A mRNA expression and modulation of angiogenic and inflammatory gene profiles; ESA enhanced neovasculogenesis in the ischemic hindlimb
Segers et al. 2011 [9]	Mouse HLI	Protease-resistant SDF-1(S4V) delivered via self-	Significantly improved perfusion by laser Doppler	N/A	Increased formation of new arterioles in ischemic tissue	Engineered SDF-1 resistant to DPP-IV/CD26 and MMP-2

		assembling peptide nanofibers	imaging at 6 weeks compared with untreated controls; nanofibers alone or wildtype SDF-1 nanofibers had no effect			cleavage; enhanced protein stability; SSDF-1(S4V) attracted smooth muscle cells without inducing mitosis, supporting vessel maturation
Kim et al. 2023 [10]	Mouse HLI	SDF-1α-eMSCs	Improved perfusion associated with vascular regeneration	Enhanced limb salvage and skeletal muscle repair	Increased vascular regeneration in ischemic tissue	Sustained paracrine SDF-1α delivery; activation of SDF-1α/CXCR4 signaling with increased ERK and Akt phosphorylation
Yin et al. 2015 [11]	Rabbit HLI	Ad-F-P-hPDMSCs	N/A	N/A	Markedly increased capillary and arteriole density in ischemic tissue	hPDMSCs survived for ≥ 4 weeks in ischemic limbs; combined cell and gene therapy enhanced angiogenesis and arteriogenesis

α-SMA: alpha–smooth muscle actin; AAV: adeno-associated virus; Ad: adenovirus; Akt: protein kinase B; ARS: acoustic-responsive scaffold; bFGF: basic fibroblast growth factor; CXCR4: C-X-C chemokine receptor type 4; DPP-IV: dipeptidyl peptidase IV; ELISA: enzyme-linked immunosorbent assay; EPC: endothelial progenitor cell; ERK: extracellular signal-regulated kinase; ESA: engineered stromal cell-derived factor-1α; FGF-2: fibroblast growth factor 2; F/P-MP: fragmin/protamine microparticle; FUS: focused ultrasound; GM-CSF: granulocyte–macrophage colony-stimulating factor; HGF: hepatocyte growth factor; HLI: hindlimb ischemia; hMYDGF: human myeloid-derived growth factor; HO-1: heme oxygenase-1; hPDMSC: human placenta-derived mesenchymal stem cell; IHC: immunohistochemistry; MMP-2: matrix metalloproteinase-2; mRNA: messenger RNA; N/A: not applicable; PBS: phosphate-buffered saline; PDGF-BB: platelet-derived growth factor-BB; rhHGF: recombinant human hepatocyte growth factor; RT-PCR: reverse transcription polymerase chain reaction; SDF-1: stromal cell-derived factor-1; SDF-1α: stromal cell-derived factor-1 alpha; SSDF-1: super-stable stromal cell-derived factor-1; S4V: Ser4→Val mutation; VEGF: vascular endothelial growth factor; VEGF-A: vascular endothelial growth factor A; VEGF165: 165-amino acid isoform of vascular endothelial growth factor; Western blot: Western blotting.

Table S5. Study timeline and experimental procedures

Study day	Procedure
Day -18	Acclimation period of between 15 and 18 days for different study cycles
Before treatment and at least twice a week thereafter	Body weight
Day 1 before surgery	Test item iPRECIO SMP-310R pump implantation just before HLI surgery (groups 1 & 2)
Day 1	HLI surgery
Day 1 (and once weekly thereafter)	Blood sampling from group 2
Day 2	IM VEGF injection after HLI surgery (group 3)
Days 1, 8, 15, 22, 29	Blood flow measurement (via Speckle Doppler imaging), conducted before and after surgery (Day 1)
Day 8 (and twice weekly thereafter)	Limb function
Twice weekly, or twice daily once necrosis appears	Necrosis evaluation
Day 29	Termination. Muscles from ipsilateral & contralateral hind limbs collected and fixed in 2.5% PFA for histology evaluation

* Blood sampling from group 2 at 7 days and 21 days after surgery was done just after blood flow measurement. HLI: hindlimb ischemia; IM: intramuscular; PFA: paraformaldehyde; VEGF: vascular endothelial growth factor.

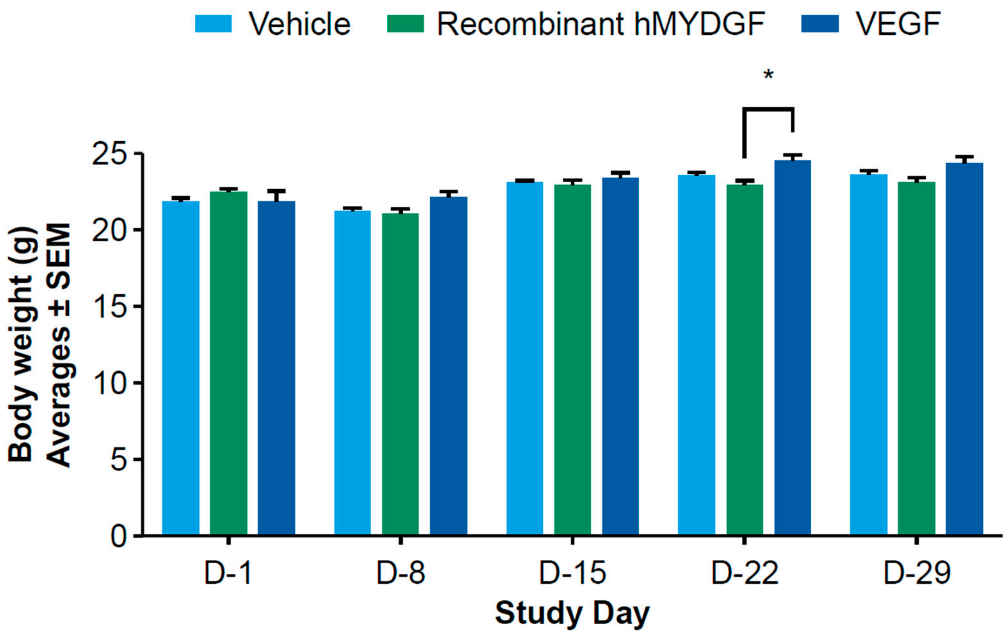

Figure S1. Body weight during follow-up. Data represent mean ± SEM. *: $p < 0.05$. D: day; hMYDGF: human myeloid-derived growth factor; SEM: standard error of the mean; VEGF: vascular endothelial growth factor. Vehicle (n = 12), hMYDGF (n = 11), VEGF (n = 13).

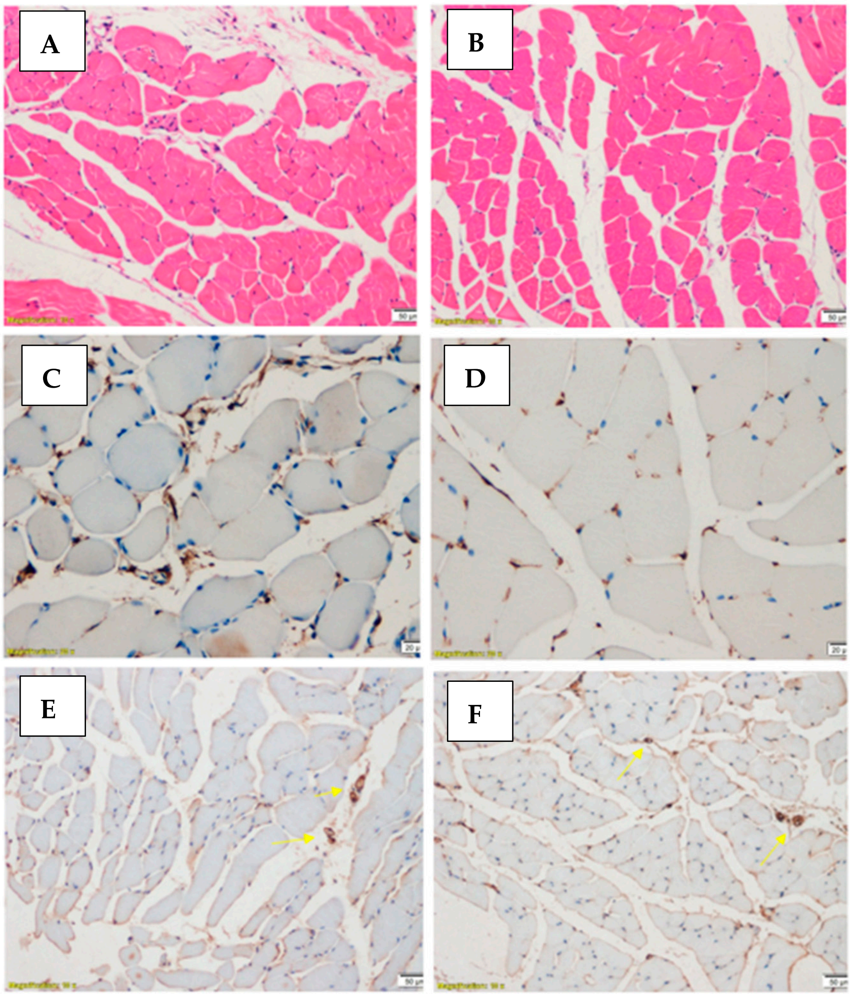

Figure S2. Representative histological and immunohistochemical images of skeletal muscle. (**A**) H&E-stained section of quadriceps muscle from the ischemic hindlimb showing mild myofiber atrophy and mild diffuse inflammatory cell infiltration. (**B**) H&E-stained section of the corresponding non-injured control hindlimb showing normal skeletal muscle architecture. (**C**) CD34 immunohistochemical staining of quadriceps muscle from the ischemic hindlimb demonstrating endothelial cells (brown). (**D**) CD34+ immunohistochemical staining of the corresponding control muscle. (**E**) α-SMA immunohistochemical staining of quadriceps muscle from the ischemic hindlimb highlighting blood vessels (brown, yellow arrows). (**F**) α-SMA immunohistochemical staining of the corresponding control muscle highlighting blood vessels (brown, yellow arrows). Nuclei are counterstained with hematoxylin (blue). Scale bars = 50 μm (A,B,E,F) and 20 μm (C,D). H&E, hematoxylin and eosin; α-SMA, alpha-smooth muscle actin; CD34, cluster of differentiation 34.

References

1. Shimpo, M.; Ikeda, U.; Maeda, Y.; Takahashi, M.; Miyashita, H.; Mizukami, H.; Urabe, M.; Kume, A.; Takizawa, T.; Shibuya, M., et al. AAV-mediated VEGF gene transfer into skeletal muscle stimulates angiogenesis and improves blood flow in a rat hindlimb ischemia model. *Cardiovasc. Res.* **2002**, *53*, 993-1001.
2. Long, J.; Wang, S.; Zhang, Y.; Liu, X.; Zhang, H.; Wang, S. The therapeutic effect of vascular endothelial growth factor gene- or heme oxygenase-1 gene-modified endothelial progenitor cells on neovascularization of rat hindlimb ischemia model. *J. Vasc. Surg.* **2013**, *58*, 756-765.e752.
3. Taniyama, Y.; Morishita, R.; Aoki, M.; Nakagami, H.; Yamamoto, K.; Yamazaki, K.; Matsumoto, K.; Nakamura, T.; Kaneda, Y.; Ogihara, T. Therapeutic angiogenesis induced by human hepatocyte growth factor gene in rat and rabbit hindlimb ischemia models: preclinical study for treatment of peripheral arterial disease. *Gene Ther.* **2001**, *8*, 181-189.
4. Morishita, R.; Nakamura, S.; Hayashi, S.; Taniyama, Y.; Moriguchi, A.; Nagano, T.; Taiji, M.; Noguchi, H.; Takeshita, S.; Matsumoto, K., et al. Therapeutic angiogenesis induced by human recombinant hepatocyte growth factor in rabbit hind limb ischemia model as cytokine supplement therapy. *Hypertension* **1999**, *33*, 1379-1384.
5. Jin, H.; Quesada, C.; Aliabouzar, M.; Kripfgans, O.D.; Franceschi, R.T.; Liu, J.; Putnam, A.J.; Fabiilli, M.L. Release of basic fibroblast growth factor from acoustically-responsive scaffolds promotes therapeutic angiogenesis in the hind limb ischemia model. *J. Control. Release* **2021**, *338*, 773-783.
6. Horio, T.; Fujita, M.; Tanaka, Y.; Ishihara, M.; Kishimoto, S.; Nakamura, S.; Hase, K.; Maehara, T. Efficacy of fragmin/protamine microparticles containing fibroblast growth factor-2 (F/P MPs/FGF-2) to induce collateral vessels in a rabbit model of hindlimb ischemia. *J. Vasc. Surg.* **2011**, *54*, 791-798.
7. de Paula, E.V.; Flores-Nascimento, M.C.; Arruda, V.R.; Garcia, R.A.; Ramos, C.D.; Guillaumon, A.T.; Annichino-Bizzacchi, J.M. Dual gene transfer of fibroblast growth factor-2 and platelet derived growth factor-BB using plasmid deoxyribonucleic acid promotes effective angiogenesis and arteriogenesis in a rodent model of hindlimb ischemia. *Transl. Res.* **2009**, *153*, 232-239.
8. Edwards, B.B.; Fairman, A.S.; Cohen, J.E.; MacArthur, J.W., Jr.; Goldstone, A.B.; Woo, J.B.; Hiesinger, W.; Woo, Y.J. Biochemically engineered stromal cell-derived factor 1-alpha analog increases perfusion in the ischemic hind limb. *J. Vasc. Surg.* **2016**, *64*, 1093-1099.
9. Segers, V.F.; Revin, V.; Wu, W.; Qiu, H.; Yan, Z.; Lee, R.T.; Sandrasagra, A. Protease-resistant stromal cell-derived factor-1 for the treatment of experimental peripheral artery disease. *Circulation* **2011**, *123*, 1306-1315.
10. Kim, J.J.; Park, J.H.; Kim, H.; Sim, W.S.; Hong, S.; Choi, Y.J.; Kim, H.J.; Lee, S.M.; Kim, D.; Kang, S.W., et al. Vascular regeneration and skeletal muscle repair induced by long-term exposure to SDF-1α derived from engineered mesenchymal stem cells after hindlimb ischemia. *Exp. Mol. Med.* **2023**, *55*, 2248-2259.
11. Yin, T.; He, S.; Su, C.; Chen, X.; Zhang, D.; Wan, Y.; Ye, T.; Shen, G.; Wang, Y.; Shi, H., et al. Genetically modified human placenta-derived mesenchymal stem cells with FGF-2 and PDGF-BB enhance neovascularization in a model of hindlimb ischemia. *Mol. Med. Rep.* **2015**, *12*, 5093-5099.
